# Peer Review “The Impact of SARS-CoV-2 Lineages (Variants) and COVID-19 Vaccination on the COVID-19 Epidemic in South Africa: Regression Study”

**DOI:** 10.2196/47143

**Published:** 2023-07-04

**Authors:** Rephaim Mpofu

**Affiliations:** 1 Division of Clinical Pharmacology University of Cape Town Cape Town South Africa

**Keywords:** COVID-19, infection, pandemic, vaccine, vaccination, epidemiology, transmissibility, health care, hospital admission, COVID-19 variants, SARS-CoV-2

*This is a peer-review report submitted for the paper “The Impact of SARS-CoV-2 Lineages (Variants) and COVID-19 Vaccination on the COVID-19 Epidemic in South Africa: Regression Study.”*

## Round 1 Review

### General Comments

This paper [1] provides an epidemiological analysis and report on the COVID-19 pandemic in South Africa and provides insight into the potential impact that various SARS-CoV-2 lineages may have had on the epidemic. Overall, this paper notes that the nonpharmaceutical interventions such as movement restrictions through lockdown measures and the evolution of the COVID-19 virus had significant impacts on the disease burden and epidemiology of disease observed in South Africa through the 3 waves that have occurred.

This manuscript is well written, comprehensive, and filled with detail. This is both a strength and a possible weakness. The strength is that the data included have been analyzed in depth, and one can be fairly certain that the results obtained are likely to be accurate. On the other hand, depending on the audience, some readers may struggle to engage with the data appropriately; the dissemination of data and reporting has not been formatted and simplified in a manner that improves readability without compromising on accuracy. The use of scientific notation for P values to the 11th power, use of 3 or 4 decimal places for proportions, etc, and extensive reporting of findings instead of picking a few of the most relevant findings with reference to the table for other findings are a few examples of this. However, this does not detract from the large amount of work that has gone into this manuscript, and the author team should be commended for it. Please find specific comments below.

### Specific Comments

#### Major Comments

1. I have not seen whether time was included as a potential confounder/covariate in any of the regression models that were conducted. Increasing immunity, the initiation of vaccination campaigns halfway through the third wave, and movement restrictions have not been discussed adequately.

2. Please provide brief details on how data used to assess movement restriction were obtained and analyzed.

3. Please comment on the appropriateness of using means and standard deviations for the description of the majority of some of these data, which may or may not have been normally distributed.

4. Please provide ethical considerations in the manuscript for the data and analysis, whether approval was required or not, and justify.

#### Minor Comments

1. “While, there is global consensus on the health risk posed by COVID-19, ground-breaking vaccine developments, and a great drive towards the vaccination of the world population against COVID-19.”

This sentence is fragmented. Please revise.

2. “emergent.” Possible typo error, consider using “emergence.”

3. National Coronavirus Command Council: A one-liner describing the National Coronavirus Command Council would be beneficial to the reader.

4. “Beta SARS-CoV-2 lineage required a half Maximal inhibitory concentration (IC50) 6 to 200 fold higher than the lineages identified in the first wave.” What reagent/antibody/method is used to test the IC 50 cited here?

5. “estimated that it was 1.29 (95%CI: 1.9601.58)).” Unsure what the confidence interval is there. Please review.

6. “period) showed significant difference at 95 % confidence interval between the respective COVID-19 epidemic periods with P values of 1.82×10-11 and 5.87×10-05 respectively.”

The author team can check submission guidelines, and the editor can confirm, but I believe that P values <.001 should be stated as such.

7. Table entries with variable names that have underscores and labeling could be cleaned up to improve readability.

8. As noted above, the use of 3 or 4 decimal places and exponential notation of extremely small P values reduces the clarity and readability. Consider reviewing.

## Round 2 Review

The manuscript has been improved based on previous reviewer comments but is still unnecessarily too long, dense, and bloated. I believe that the adage “simpler is better” would have suited the objectives of this paper well. The average reader may find it difficult to read to the end, and some readers may have difficulty fully engaging with the content as a result. Five pages on the virology of SARS-CoV-2 as an introduction is likely unnecessary for a manuscript whose data focus on the epidemiology and statistics of COVID-19 rather than its virology.

There are many statistical tests conducted here; however, the authors do not appear to have performed any adjustments for the multiple tests conducted. The familywise error rate is bound to be higher than 0.05, so some of your conclusions based on the statistical probability may be inaccurate.

Finally, there are some statements that have been made based on the Discussion and Conclusion sections that I do not believe are adequately supported by the data presented, and these may need to be reconsidered/softened. Please see specific comments below.

1. Methods: Many hypothesis tests are conducted in this paper. Was adjustment for multiple testing performed? Otherwise, the possibility of making type 1 errors is quite high. This should either be reviewed or listed as a key limitation.

2. South Africa community mobility data: How is movement in these data measured? Kilometers? Significant movement out of the house? The number of people in an area? Please describe.

3. “The mean daily positive COVID-19 tests in South Africa’s first and second COVID-19 epidemic wave had no statistically significant difference.”

Please report the data and P values or reference the table where these data can be found.

4. Please insert a legend for the figures (eg, Figures 7 and 8).

5. Table 1: The maximum COVID-19 hospitalized intensive care unit percentage of 7 and 814.1 is unclear.

6. Discussion: “The values of the Pearson and Spearman Correlation Coefficients obtained between the daily COVID-19 tests and cases in this study indicated a strong positive correlation between daily COVID-19 tests and cases with more than 95 % confidence in the four COVID-19 epidemic waves in South Africa.”

Please review this interpretation of your correlation significance and 95% confidence intervals. It is technically incorrect to say that “there is more than 95% confidence.”

As a suggestion, you may leave the 95% confidence part out altogether and just say that testing was significantly related to case incidence in the 4 COVID-19 waves.

Consider also reviewing the American Statistical Association papers on P values and moving toward more conservative reliance on statistical significance overall (Wasserstein RL, Schirm AL, Lazar NA. Moving to a world beyond “*P*<0.05”. *Am Statistician*. 2019;73(sup1):1-19. doi:10.1080/00031305.2019.1583913).

7. These data, as presented, do not allow you to make this conclusion as you have not made a relationship of causality, but rather have demonstrated an association, as you rightly say in the following lines. Please revise to describe this as a significant association rather than a causal relationship.

8. “To understand the causality of relationships between two or more variables, statistical theory must be applied.” Text like this is unnecessary and contributes to the bloating of your manuscript. Consider removing.

9. “Daily COVID-19 tests in South Africa were observed to be normally distributed while the daily COVID-19 cases were positively skewed with a lognormal distribution (Galton distribution).”

I do not recall the data distributions being assessed or described in the Results, so it is surprising that they are now included in the Discussion. Consider including or revising the need to discuss the data distributions (a similar comment applies to the following paragraph).

10. I have reservations about the use of the word “confounder” in this discussion. While the movement is most likely a potential contributing factor in the detection rate of COVID-19, this was not analyzed or demonstrated using appropriate statistical methods such as multiple regression or interaction tests.

Showing that there was a correlation between population movement and COVID-19 detection does not automatically demonstrate that movement is a significant confounder. The messaging may have to be altered to suggest a possible confounding effect, or alternatively, this would need to be demonstrated by conducting appropriate data analysis.

11. “The values of the Spearman Correlation Coefficients obtained between the daily cumulative COVID-19 vaccinated people and change in daily COVID-19 cases in the half period of the third and fourth COVID-19 epidemic wave in this study indicated a low correlation between the daily cumulative COVID-19 vaccinated people and change in daily COVID-19 cases with this correlation statistically insignificant.”

This statement should be reconsidered. If vaccination does indeed have a significant effect on daily infection rates, there is bound to be a lag between exposure and effect, and this would need to be demonstrated in a robust time series analysis. Correlating the vaccination rate with the COVID-19 case rate without adjustment for time periods would not adequately demonstrate the effect of vaccination if such an effect existed. This is particularly important because the statement “These results suggest that COVID-19 vaccines administered in South Africa had no significant effect on the transmission of COVID-19” would be a controversial conclusion to come to without solid evidence to support this statement that may be seen as inflammatory in the politically charged topic of vaccines and vaccine hesitancy in South Africa.

12. “This result can be explained by the percentage of the population per age group who had received at least one dose of the COVID-19 vaccine by the end of the fourth COVID-19 epidemic wave.”

This statement appears to contradict your earlier statement that vaccines did not appear to have an impact on COVID-19 transmission in South Africa. Please review and reconcile. Also, natural immunity and potentially reduced virulence of the Omicron variant are important factors to consider in the reduced mortality in the fourth wave.

13. “showed statistical significant indifferences at 95 % confidence.” Unusual wording and terminology such as indifference at 95% confidence. Please revise.

14. “While COVID-19 vaccines administered in South Africa had no significant effect on the transmission of COVID-19 within the South African population.”

Again, this statement is not supported by the data provided and should be reviewed and reconsidered.

15. Table A. 1: Consider formatting these large sums of square and mean square values including thousand separators for readability.

## Round 3 Review

Thank you for the review comments and revisions.

### Comments

1. Table 8: Consider having the cumulative COVID-19 death risk ratio value for the reference group as “Ref” for reference. It may be confusing to have a risk ratio for the reference category.

2. Table 9: Case-fatality rate is abbreviated as “CRF” at times (and in subsequent text) and as “CFR” at times.
